# Subcutaneous infusion of high-dose benzathine penicillin G is safe, tolerable, and suitable for less-frequent dosing for rheumatic heart disease secondary prophylaxis: a phase 1 open-label population pharmacokinetic study

**DOI:** 10.1128/aac.00962-23

**Published:** 2023-11-16

**Authors:** Joseph Kado, Sam Salman, Thel K. Hla, Stephanie Enkel, Robert Henderson, Robert M. Hand, Adam Hort, Madhu Page-Sharp, Kevin Batty, Brioni R. Moore, Julie Bennett, Anneka Anderson, Jonathan Carapetis, Laurens Manning

**Affiliations:** 1 Wesfarmers Centre for Vaccines and Infectious Diseases, Telethon Kids Institute, University of Western Australia, Perth, Western Australia, Australia; 2 Medical School, University of Western Australia, Perth, Western Australia, Australia; 3 Clinical Pharmacology and Toxicology Unit, PathWest, Western Australia, Australia; 4 Department of Infectious Diseases, Fiona Stanley Hospital, Perth, Western Australia, Australia; 5 Medical Imaging Department, Perth Children’s Hospital, Nedlands, Western Australia, Australia; 6 Department of Infectious Diseases, Royal Perth Hospital, Perth, Western Australia, Australia; 7 Western Australian Country Health Service, Perth, Western Australia, Australia; 8 Curtin Medical School, Curtin University, Bentley, Western Australia, Australia; 9 Department of Public Health, University of Otago, Wellington, New Zealand; 10 Te Kupenga Hauora Maori, University of Auckland, Auckland, New Zealand; 11 Department of Infectious Diseases, Perth Children’s Hospital, Perth, Western Australia, Australia; Providence Portland Medical Center, Portland, Oregon, USA

**Keywords:** benzathine penicillin G, subcutaneous infusions, secondary antibiotic prophylaxis, rheumatic heart disease, population pharmacokinetics

## Abstract

Since 1955, the recommended strategy for rheumatic heart disease (RHD) secondary prophylaxis has been benzathine penicillin G [BPG; 1.2 MU (900 mg)] injections administered intramuscularly every 4 weeks. Due to dosing frequency, pain, and programmatic challenges, adherence is suboptimal. It has previously been demonstrated that BPG delivered subcutaneously at a standard dose is safe and tolerable and has favorable pharmacokinetics, setting the scene for improved regimens with less frequent administration. The safety, tolerability, and pharmacokinetics of subcutaneous infusions of high-dose BPG were assessed in 24 healthy adult volunteers assigned to receive either 3.6, 7.2, or 10.8 MU (three, six, and nine times the standard dose, respectively) as a single subcutaneous infusion. The delivery of the BPG to the subcutaneous tissue was confirmed with ultrasonography. Safety assessments, pain scores, and penicillin concentrations were measured for 16 weeks post-dose. Subcutaneous infusion of penicillin (SCIP) was generally well tolerated with all participants experiencing transient, mild infusion-site reactions. Prolonged elevated penicillin concentrations were described using a combined zero-order (44 days) and first-order (*t*_1/2_ = 12 days) absorption pharmacokinetic model. In simulations, time above the conventionally accepted target concentration of 20 ng/mL (0.02 µg/mL) was 57 days for 10.8 MU delivered by subcutaneous infusion every 13 weeks compared with 9 days of every 4-weekly dosing interval for the standard 1.2 MU intramuscular dose (i.e., 63% and 32% of the dosing interval, respectively). High-dose SCIP (BPG) is safe, has acceptable tolerability, and may be suitable for up to 3 monthly dosing intervals for secondary prophylaxis of RHD.

## INTRODUCTION

Acute rheumatic fever (ARF) results from an abnormal autoimmune response to inadequately treated throat or skin infections with Group A Streptococcus (1) (*Streptococcus pyogenes*; GAS), which presents with joint, cardiac, skin, and neurological manifestations. Severe or repeated episodes of ARF may result in chronic damage to heart valves called rheumatic heart disease (RHD). ARF and RHD predominantly affect children and young adults in resource-limited settings, but marginalized populations in high-income countries are also affected (2, 3). RHD is estimated to affect over 40.5 million people globally and cause 306,000 deaths annually (4).

There is no specific treatment for ARF. However, the mainstay of current therapy, benzathine penicillin G (BPG), which is a long-acting formulation, is directed at preventing the colonization or infections with *S. pyogenes* to prevent the recurrence of ARF and worsening of RHD. Despite being used since the 1950s for secondary prophylaxis of ARF and RHD, BPG has been shown to be superior to other forms of penicillin and other antibiotics (5). Current guidelines recommend BPG 1.2 million units (MU) delivered by deep intramuscular (IM) injection every 3–4 weeks for a minimum of 5 years to prevent progression to or worsening of RHD (6–8).

Although it has proven efficacy, adherence to secondary prophylaxis is low in many RHD-prevalent communities (9, 10). There are many patient and provider factors that present barriers to adherence (10), but the frequency and mode of delivery are also inconvenient and painful. A consultation with global RHD experts on the ideal characteristics required for an alternate long-acting penicillin preparation supported subcutaneous (SC) (rather than IM) administration, an extended duration of protection (3–6 months), and cold-chain independence, while maintaining affordability (11).

Subcutaneous delivery of BPG is safe and potentially advantageous (12). Recent pharmacokinetic (PK) studies in adolescents with ARF have indicated that subcutaneous delivery has been inadvertently provided to overweight individuals without apparent side effects but with a favorable absorption profile (13, 14). These findings were confirmed in a phase 1 clinical randomized cross-over trial of SC BPG, which confirmed delayed absorption and resulted in lower peak penicillin concentrations and prolonged duration of effect, comparable pain scores, and no serious adverse events (12). Simulations from this trial predicted that, if safe and tolerable, subcutaneous infusion of high-dose benzathine penicillin G (SCIP RHD) could provide adequate penicillin concentrations for up to 3 months. Here, we report the safety, tolerability, and PK of SCIP RHD in a phase 1 trial.

## RESULTS

### Participants

**Fig 1:**
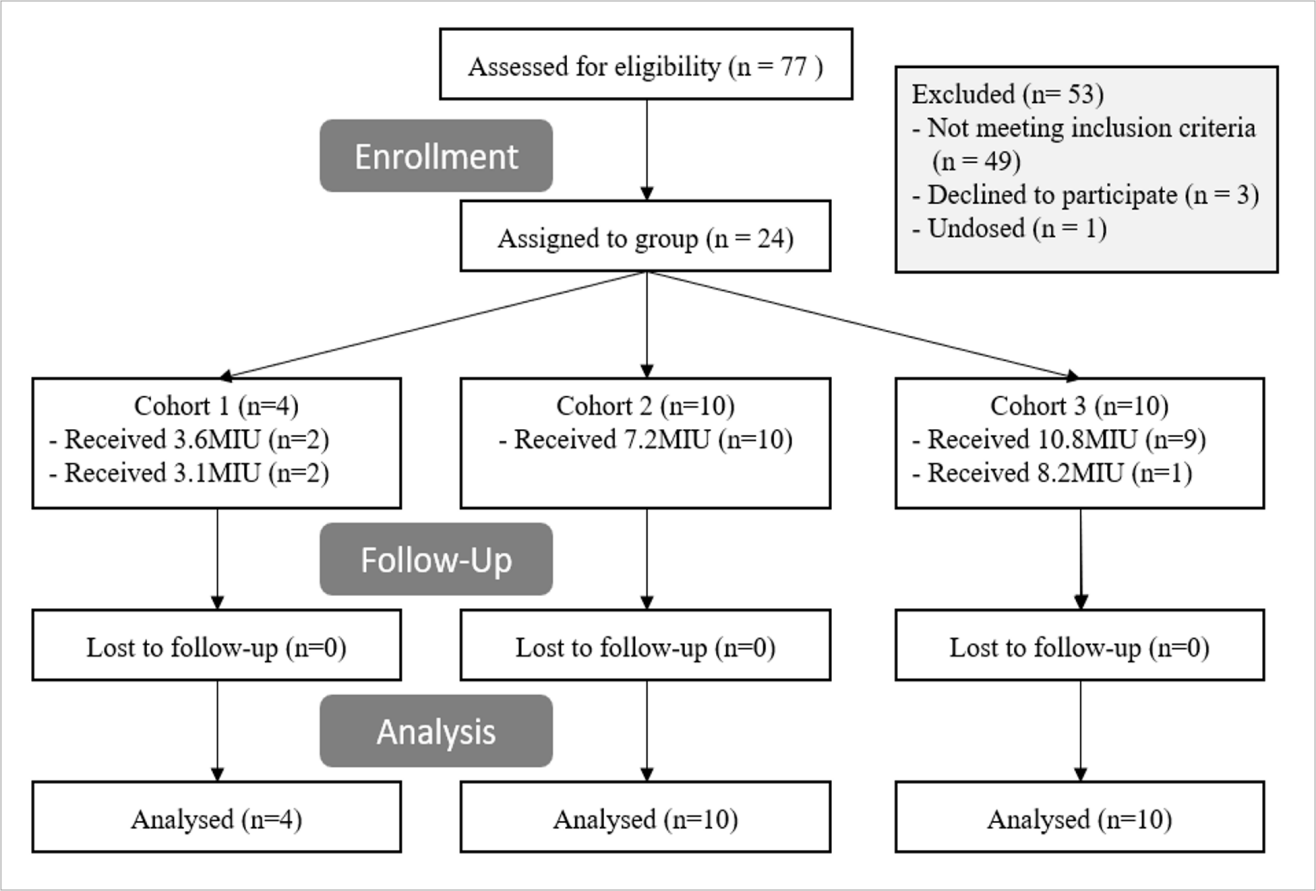
Consort diagram for trial participants.

Seventy-seven adults were screened for eligibility, and 24 eligible participants were recruited; four were females. The median (range) screening age and body mass index (BMI) were 26.9 (18.0–54.1) years and 25.1 (21.9–34.0) kg/m^2^, respectively. Eight participants identified as European, eight Asian, four Hispanic/Latino, two African, and two of mixed race. All participants completed the study. The study Consort diagram is shown (Fig. 1).

### Study infusion

Three participants did not receive the planned dose of BPG. The first two participants in cohort 1 received 3.1 MIU because ~1 mL infusate failed to progress through the flow control extension tubing, while one participant in cohort 3 received 8.2 MIU as the infusion after a period of cessation due to pain. All other participants received planned dosages of 3.6 MIU (*n* = 2), 7.2 MIU (*n* = 10), and 10.8 MIU (*n* = 9).

### Pain scores

Reported numerical rating scale (NRS) pain scores ranged from 0 to 8 with the greatest intensity recorded during the infusion, but the median (range) pain scores were 2.5 (0–6), 3 (0–8), and 2.5 (0–6) for cohorts 1, 2, and 3, respectively. Moderate pain (NRS 4–7) was not experienced beyond 2 days, and no pain was reported beyond 7 days. The median (range) of pain scores for each cohort is shown (Table 1). While all Day 3 pain scores were in the mild range (0–3), there were differences noted between dosing cohorts 1 and 3 (*P* = 0.007) and cohorts 2 and 3 (*P* < 0.05; Fig. S1: Comparison of median reported NRS pain scores by time post-infusion and dosing cohort). There was no significant difference in reported pain scores between the ideal and high BMI groups at any sampling time point (Table S1).

**TABLE 1:** Median (range) reported numeric pain scores according to dosing cohort following subcutaneous infusion of benzathine penicillin G

Cohort	Dose (MIU)	0 h (range)	2 h (range)	6 h (range)	12 h (range)	1 day (range)	2 days (range)	3 days (range)	5 days (range)	7 days (range)
1 (*n =* 4)	3.6	2.5 (0–6)	0.5 (0–1)	0 (0–1)	0 (0–1)	0 (0–2)	0.5 (0–2)	0 (0–0)	0 (0–0)	0 (0–0)
2 (*n =* 10)	7.2	3 (0–8)	0 (0–0)	0.5 (0–2)	0 (0–1)	1 (0–4)	0.5 (0–2)	0 (0–2)	0 (0–0)	0 (0–0)
3 (*n =* 10)	10.8	2.5 (0–6)	0 (0–6)	0.5 (0–1)	1 (0–3)	2 (0–6)	2 (0–6)	1.5 (1, 2)	0 (0–2)	0 (0–1)

### Modified Skindex-16 scores

Normalized overall Skindex-17 scores ranged from 0 to 61 with the highest scores reported in the first 2 days after infusion. Scores decreased thereafter with only four participants reporting scores beyond Day 14. The highest normalized domain (symptoms) score was 75 reported by a cohort 2 participant on Day 2. The normalized domain and overall Skindex-16 scores for the first 7 days by cohort and all cohorts combined are provided (Table S2).

For a between-cohort comparison of normalized overall Skindex scores by day, the only significant difference was noted on Day 3 between cohorts 1 and 2 (*P* = 0.007) (Table S3). There was no significant difference in the normalized overall scores between the high and ideal BMI groups (Table S4).

### Adverse events

There were 101 adverse events reported during the study with no serious adverse events (Table S5). All participants experienced some infusion-site reaction including erythema, swelling, tenderness/pain, and pigmentation ranging in duration from less than 1 day (pain) to 217 days (hyperpigmentation). There were two off-study referrals for post-infusion hyperpigmentation, but no treatment was required, as they resolved spontaneously. Ultrasound changes were recorded in all participants post-infusion and were detectable between 4 and 9 months. Non-specific hyperechoic changes were noted in most participants, but two participants developed a loculated collection that resolved by 6.5 and 8.3 months.

### Pharmacokinetic modeling

From the 24 individuals, there were 400 samples included in the PK analysis. The fraction of samples below the limit of quantification (LOQ) (BLQ) was low (11%), those measured between the LOQ and limit of detection (LOD), *n* = 12 (3.0%), were included at their measured amount, and those below the LOD, *n* = 31 (7.7%), were excluded from the analysis. The highest benzylpenicillin concentration was less than 300 ng/mL. Plasma versus dried blood spot (DBS) concentrations were highly correlated (Fig. S2) and compared in early modeling with an addition parameter used to estimate the difference between the two. A bootstrap (*n* = 1,000) performed resulted in a median of 0.990 for this parameter with a 95% confidence interval (CI) of 0.897–1.08. Further modeling proceeded using DBS data only.

**Fig 2:**
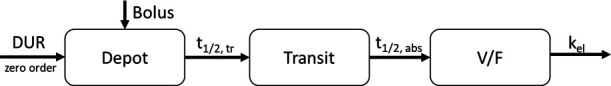
Structure of the pharmacokinetic model. The best model consisted of two absorption paths, one a bolus into the depot compartment and the other a zero-order process into the same depot modeled as the duration. Absorption from this depot compartment was through a transit compartment with corresponding transit half-life (*t*_1/2, tr_) before absorption (*t*_1/2, abs_) into the main compartment (V/F). *k*_el_ represents the elimination rate constant.

Visual inspection of the population data demonstrated a change in the terminal phase occurring after 42 days, with a steeper decline in inter-individual variability (IIV) estimated after this point. Several models with zero-order absorption were assessed with the best model consisting of two absorption paths, one a bolus into the depot compartment and the other a zero-order process into the same depot modeled as the duration (DUR). Absorption from this depot compartment was through a transit compartment with corresponding transit half-life (*t*_1/2, tr_) before absorption (*t*_1/2, abs_) into the main compartment. The model structure is provided (Fig. 2). A ratio parameter (RATIO) was included to estimate the ratio of dose considered to be input into the depot compartment as a bolus relative to the zero-order input rate.

The IIV was estimated on volume (V/F) as well as separate IIV values for SC administration for DUR, *t*_1/2, abs_, and RATIO. While adjusted body weight (ABW) resulted in a slightly lower objective function (OFV) than weight (WT) (∆OFV−0.696) as the size covariate for allometric scaling, there were no other improvements in terms of goodness-of-fit (GOF) or variability terms. Therefore, given this improvement was only marginal and to allow easy comparison with previous publications, WT was chosen as the optimal size parameter for allometric scaling. Several significant covariate relationships with DUR were identified including waist circumference, hip circumference, and brachioradialis US SC fat measurement; however, BMI with a power relationship resulted in the largest change in OFV (−6.105). No other significant covariate relationships were identified.

**Fig 3:**
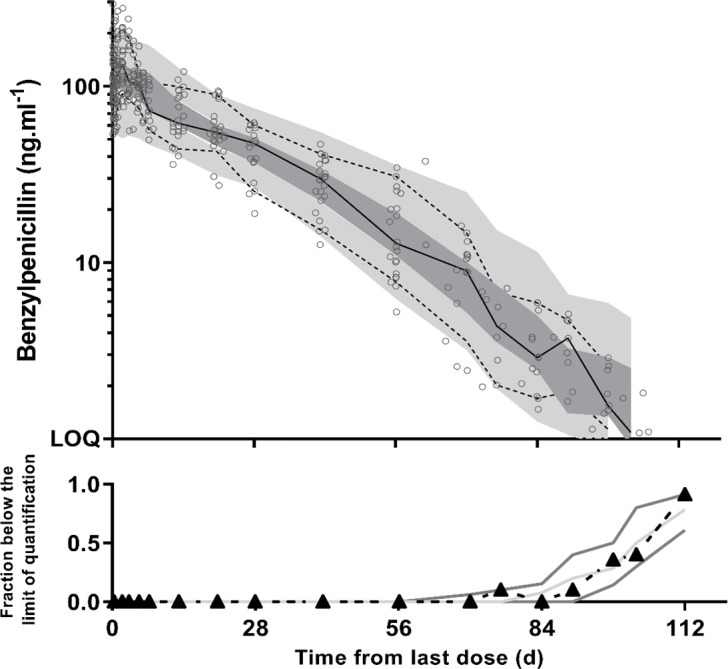
Prediction-corrected visual predictive checks for plasma benzylpenicillin G concentrations (ng/mL on log10 scale). Observed 50th (solid line) and 10th and 90th (dotted lines) percentiles within their simulated 95% CI (gray shaded areas) are shown with overlying data points (ο, *n* = 400).

The final model parameter estimates and the bootstrap results are presented (Table 2). The GOF plots did not show any evidence of bias (Fig. S3). The raw data points and prediction-corrected visual predictive check (pcVPC) plots are shown (Fig. 3). The actual 10th, 50th, and 90th percentiles of observed data as well as the fraction of BLQ data mostly fell within their respective 95% confidence intervals, demonstrating suitable predictive performance of the model. The pcVPC also did not suggest any bias from the exclusion of data below the limit of detection.

**TABLE 2:** Final population pharmacokinetic estimates and bootstrap results following subcutaneous infusions of benzathine benzylpenicillin G

Parameter^*b*^	Mean	RSE (%)^*a*^	Bootstrap median (95% CI)
Objective function value	521.69		536.929 (−603.549–−476.86)
Common structural model parameters			
*k*_el_ (h^−1^·70 kg^−1^)	1.32		Fixed
V/F (L·70 kg^−1^)	42.8	4%	42.8 (40–46.1)
*t*_1/2, abs_ (days)	11.8	4%	11.8 (10.8–12.8)
*t*_1/2, tr_ (hours)	0.702	13%	0.69 (0.519–0.873)
DUR (hours)	1,062	6%	1,055 (832–1,250)
RATIO	2.54	2%	2.47 (1.66–4.65)
Exponent of power BMI relationship on DUR	1.13	28%	1.11 (0.21–2.22)
Common variable model parameters (shrinkage%)			
IIV in V/F	15 (9)	17%	15 (10–20)
IIV in DUR	22 (14)	11%	22 (10–33)
IIV in *t*_1/2, abs_	12 (26)	23%	11 (6–18)
IIV in RAT	88 (16)	23%	85 (52–134)
RV (%)	24 (10)	5%	24 (21–26)

^*a*^ Calculated from bootstrap.

^*b*^ RSE, relative standard error; *k*_el_, elimination rate constant; V/F, relative volume of distribution; *t*_1/2, tr_, transit compartment half-life; *t*_1/2, abs_, absorption half-life; RATIO, the relative amount of drug assigned to bolus over zero-order input into the depot compartment; RAT, ratio between different absorption paths; IIV, inter-individual variability; RV, residual variability. IIV and RV are presented as 100% × √(variability estimate).

The population estimate for RATIO corresponds to 28% of the dose passing through the zero-order absorption pathway; notably, this parameter had the highest IIV (88%). The average duration of this process was around 44 days with the effect of BMI resulting in a range of 38–63 days for the lowest (15) and highest (16) BMI in the study, respectively.

**Fig 4:**
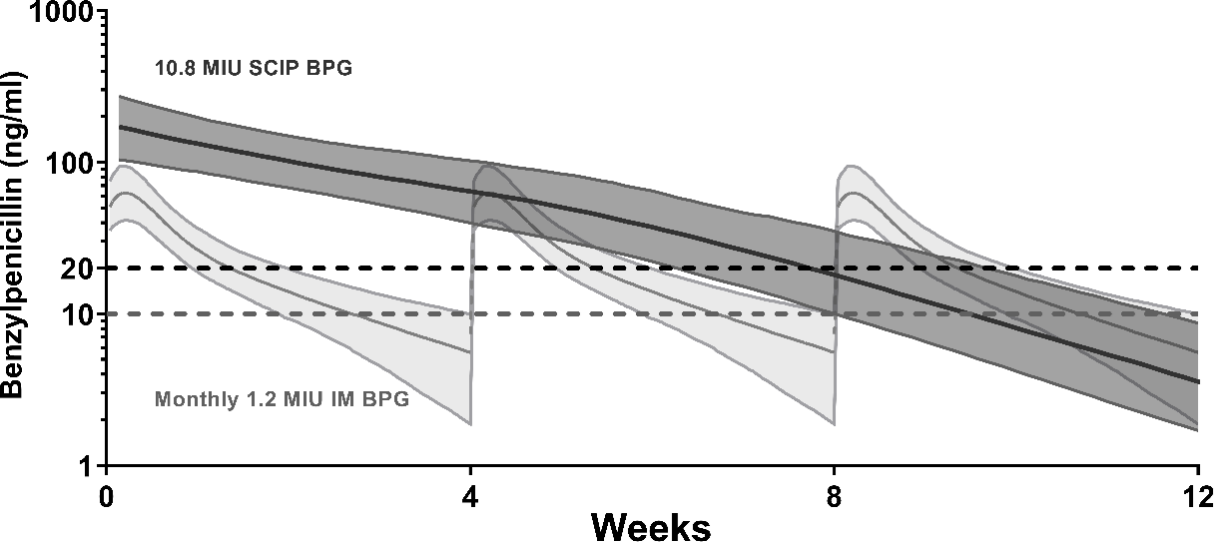
Simulations of the pharmacokinetic model comparing high-dose subcutaneous infusions of benzathine penicillin G (10.8 MIU, dark gray) with intramuscular injection (1.2 MIU, light gray). The shaded region represents median with 90% simulation interval.

Simulations demonstrated a longer proportion of time between doses above the threshold of 20 ng/mL for the 13-weekly SCIP 10.8 MIU dose [median 63%, 90% simulation interval (SI) (51%–77%)] compared to standard 4-weekly IM 1.2 MIU doses [median 39% (27%–56%)] (12). However, the higher dose resulted in more absolute time below this threshold, median of 4.86 vs 2.43 weeks for the SCIP and IM doses, respectively. For a lower threshold of 10 ng/mL, the difference was less pronounced with a median of 75% (64%–91%, 90% SI) for the SCIP dose and 67% (47%–99%, 90% SI) for the standard IM dose (Fig. 4).

## DISCUSSION

Subcutaneous infusion of high-dose BPG (SCIP) is tolerable and safe. Despite all participants experiencing some degree of infusion-site reaction, they all resolved spontaneously with no serious adverse outcomes. While pain is a subjective experience, the maximum reported pain at injection was severe (NRS 8), requiring analgesia, in only one participant; moderate pain (NRS 4–7) was experienced by another 11 participants in the first 2 days; nevertheless, the median cohort pain scores were all in the mild (1–3) category. Although we did not directly compare SCIP with intramuscular BPG, similar median or mean pain scores have been reported in other studies involving an intramuscular dose of 1.2 MIU of BPG in healthy adults (12) and pediatric or adult populations receiving ARF secondary prophylaxis doses (17–20), with moderate–severe pain at the time of injection, which resolves over 48 hours. This suggests that pain of SCIP may not be a limiting factor in people already receiving regular BPG injections.

Participant experience of the infusion-site reaction indicated by modified Skindex-16 scores was variable, ranging from very little (zero) to fairly severe (75) “bother,” but severity categories have not been defined in literature. Nonetheless, the median scores all fall within the mild–moderate (0.8–12.5) categories, and the impact resolved within 3 days. Hyperpigmentation persisted beyond the first week and was reflected by scores in the emotional domain. As described in literature, hyperpigmentation was more common in darker-skinned participants in the study and carried some emotional stress, worsened by uncertainty and delayed resolution (21).

Despite the high dose delivered in SCIP, the peak concentration was well below concentrations achieved with standard doses of intravenous penicillin G. Simulations from the PK model indicate that penicillin concentrations above the PK/pharmacodynamics (PD) target for *S. pyogenes* (20 ng/mL) from SCIP compared to 4-weekly standard IM doses were between 26.5–40.0 weeks and 14.0–29.1 weeks for a 12-month period, respectively. A lower target of 10 ng/mL would provide 33.3–47.3 weeks above the target for SCIP and 24.4–51.5 weeks for the standard IM dose. Time >20 ng/mL suggests that SCIP is able to deliver an acceptably increased dosing interval for secondary prophylaxis of ARF (11) and has important clinical implications. Assuming the higher PK/PD target, a reduction from 13 clinic visits to four visits per annum would be an economic benefit for the health provider, patient, and family. A lower target will result in a greater dosing interval and highlights the need to determine the minimum effective concentration of penicillin required to prevent *S. pyogenes* infections. This PK model also provides a platform for tailoring SCIP dosing for smaller children and adolescents.

While the participants found SCIP safe and tolerable, adults and children living with RHD will determine the acceptability of SCIP as they will have had experience with standard IM injections and an expectation of what is acceptable pain, erythema, or inconvenience related to function and emotional burden. Adolescents may experience difficulty with being unable to expose their abdomen for prolonged periods after SCIP and coping with the possible distress should hyperpigmentation develop. Less exposed sites for SCIP should be investigated along with the feasibility of earlier topical treatment for hypermelanosis.

Younger patients with more pliant subcutaneous tissue may tolerate SCIP better than older patients, but this should be balanced with closer attention to the spring-loaded pump and flow control adjustment to prevent excessive pressure and bruising at the site.

The limitations of this study include that the study was conducted in healthy adults without RHD, and women were under-represented. While we were able to deliver SCIP using the spring-loaded pump and a variable flow control device, there was limited scope for fine-controlling the flow of BPG because it only flowed with the highest settings. An improved flow control device is needed. While the sample size was adequate for PK modeling, it restricted subgroup analysis. Lastly, most people with RHD live in resource-constrained countries where pre-suspended BPG is not available and therefore will not have access to SCIP. There is an urgent need to explore SCIP using reconstituted powdered BPG formulations or improve access to pre-filled BPG preparations if SCIP is to be widely implemented.

## MATERIALS AND METHODS

### Study design

We conducted a phase 1 open-label study using three cohorts with increasing doses of BPG, with the primary objective of assessing the safety and tolerability of SCIP. The secondary objectives were to characterize the PK, including the time above the current accepted pharmacological surrogate for protection against *S. pyogenes* and any potential effect of body composition on absorption profiles. The protocol was approved by the Bellberry Human Research Ethics Committee (2020-12-1348) and prospectively registered (Australian and New Zealand Clinical Trial Registry; ACTRN12621000135819). All participants provided written informed consent.

### Participants

Twenty-four healthy participants were recruited into the study from eligible adults aged 18–65 years, without significant co-morbidities. Full eligibility/exclusion criteria are provided (Table S6). To explore the effect of body composition, 12 participants with an ideal BMI (20–24.9 kg/m^2^) and 12 with higher BMI (25–34.9 kg/m^2^) were enrolled. The sample size calculation for comparison between ideal weight and overweight/obese participants, based on data from our recent study comparing IM and SC injections of 1.2 MU BPG, which demonstrated a difference of ~50% in the absorption characteristic (*K*_a1_) (12), a clinically meaningful difference between different body composition, with 80% power and α 0.05, would require ~10 participants in each group (12).

### Study intervention

Participants received a single SC infusion of 3.6 (2,700 mg, 9 mL), 7.2 (5,400 mg, 13.8 mL), or 10.8 MIU (8,100 mg, 20.7 mL) of BPG (Bicillin L-A, Pfizer, Sydney, Australia) delivered under ultrasound guidance. To achieve sustained plasma penicillin concentrations of 73% and 99% for a 3-month period above target concentrations of 20 ng/mL and 10 ng/mL, respectively, we chose a maximal dose of 10.8 MIU BPG (nine vials) (12). To ensure safety and tolerability, we incrementally increased the infusion doses. Because 1.2 and 2.4 MIU are the current standard treatment for RHD (6, 8) and infectious syphilis (15, 22), respectively, the starting dose was 3.6 MIU for cohort 1 (*n* = 4), increasing to 7.2 MIU for cohort 2, which is equivalent to the cumulative dose for late latent syphilis (23, 24) before cohort 3 (10.8 MIU; *n* = 10). Study progression to a higher-dose cohort was overseen by a safety monitoring committee (SMC).

Assignment to treatment was by recruitment order and BMI group ensuring equal BMI representation in each cohort. An independent SMC was appointed to review the safety of study procedures and all adverse events 72 hours after dosing, to approve progression to the next cohort. In particular, skin necrosis at the injection site, which has been reported following SC infusion of other antibiotics (25, 26), was a specific possible serious adverse event for consideration.

### Study infusion

Prior to infusion, participants underwent an ultrasound examination (Acuson S3000 and Acuson 18L6 probe, Siemens Medical Solutions, Malvern, USA) to assess SC fat using a validated protocol (27).

The prescribed BPG dose was transferred to a 30 mL syringe (JMS Co. Ltd, Hiroshima, Japan) and delivered into a single injection site on the lower anterior abdomen over a period of up to 30 minutes using a spring-driven syringe infusion pump (Springfusor30, Go Medical Industries Pty Ltd., Subiaco, Australia), a variable flow control device (VersaRate Plus, EMED Technologies, El Dorado Hills, California, USA) and a 22 G SC catheter (BD Saf-T-Intima, BD Medical, Mississauga, ON, Canada). Two milliliters of 1% lignocaine was used to prime the system and provide local anesthesia prior to commencing the infusion.

### Pharmacokinetic samples

Pharmacokinetic samples consisted of serial dried blood spots and venous samples. DBSs were collected 2, 6, 12, 24, 48, and 72 hours pre-dose and 5, 7, 14, 21, 28, 42, 56, 70, 84, 98, and 112 days post-dose, and two venous blood samples were collected at 12 hours and 14 days post-dose. Three to five drops of mixed capillary blood from a fingerprick were individually spotted directly on filter paper cards (Whatman 903 Protein Saver Cards, GE Healthcare, Parramatta, NSW, Australia) to allow the blood to be drawn by capillary action and distributed evenly on the DBS card. The card was air-dried at room temperature for 1.5 hours and stored at <12°C for at least 3 hours to dry, placed in an airtight foil envelope with a single desiccant sachet, and stored at −80^o^. Venous samples for internal validation of the DBS assay, collected concurrently with DBS samples at 12 hours and 14 days post-dose, were separated within an hour of collection into aliquots of whole blood, plasma, and cell pellets and stored separately at −80^o^ to prevent penicillin instability. A validated liquid chromatography–mass spectroscopy (LC-MS) assay was used to assay penicillin concentrations (28). The full schedule of assessments is provided (Table S7).

### Safety monitoring

Safety and tolerability were assessed using the numerical rating scale for pain (29), complemented by semi-structured qualitative interviews of the infusion experience, monitoring for infusion-related adverse events including ultrasound examination of the infusion site, and a modified Skindex-16 score to measure the effect of infusion-site skin conditions on participants’ quality of life (30). During the infusion, participants determined when the rate of infusion should be slowed or paused, titrated against their level of comfort.

The NRS with scores from 0 to 10 was used for its ease of application and validity in assessing acute and change in pain over time, with 0 and 10 representing “no pain” and “worst pain imaginable,” respectively. The minimally clinically important differences (MCIDs) for moderate pain (NRS 4–7) and severe pain (NRS 8–10) are 1.3 and 1.8, respectively, but there is no MCID for mild pain (NRS 1–3) (31).

Semi-structured qualitative interviews were conducted just prior to and during the infusion process and repeated at 2 hours and 7 days post-infusion to better understand the SCIP experience. Interviews were recorded, transcribed verbatim, and analyzed using thematic analysis. The qualitative assessment of the volunteer experience of SCIP has been reported separately (32).

We used a modified Skindex-16 questionnaire (30) (Table S8) comprising 16 self-assessment items to evaluate the impact on the quality of life, associated with the infusion-site skin condition. Rather than the frequency of symptoms, Skindex-16 covers three main domains: symptoms, emotions, and function comprising four, seven, and five items, respectively. The instrument header inquires, “*How often have you been bothered by*” with each item comprising seven boxes (0–6) anchored by “never bothered” and “always bothered.” The Skindex score has been validated for comparison over time, between and pre-/post-treatments (33). The three domain scores are normalized on a scale of 0–100 representing “no bother” and “always bothered,” respectively. The overall score averages the three normalized scores. The MAPI Research Trust Fund and the author granted permission for adaptation and use of the Skindex-16 instrument.

### Population pharmacokinetic analysis

Log_e_ plasma concentration–time data sets for penicillin were analyzed by nonlinear mixed-effects modeling using NONMEM (v 7.2.0, ICON Development Solutions, Ellicott City, MD, USA) with an Intel Visual Fortran 10.0 compiler. The first-order conditional estimation with interaction (FOCE with INTER) method was used. The minimum value of the objective function and visual predictive checks were used to choose suitable models during the model-building process. A significance level of *P* < 0.01 was set for comparison of nested models. Allometric scaling for size was employed *a priori*, with an exponential of 1 for volume (V) and 3/4 for clearance (CL) terms (16). Residual variability (RV) was estimated as an additive error for the log-transformed data.

The principal structure of the pharmacokinetic modeling was based on our previous published population of PK models (13, 14, 34) where the elimination rate was fixed and the observed PK curve described using various absorption models. This approach is consistent with the much slower absorption of the BPG salt (days), relative to the short elimination half-life of benzylpenicillin (<1 hour). When assessed against the model for standard doses administered SC in healthy volunteers, the present data demonstrated significant bias at later time points that were not encompassed in the earlier model (follow-up to 42 days vs 112 days here). Therefore, alternative absorption models were assessed, which included paths of zero-order absorption, in sequence or in parallel to first-order absorption, with multiple simultaneous absorption processes (up to four) and transit compartments with ratio parameters for parallel absorption parameters.

Inter-individual variability as well as correlations between IIV terms was then evaluated for each parameter. The IIV was exponentially modeled for all parameters. Given the wide range of participant sizes, the most appropriate measure for allometric scaling (affecting volume and clearance terms) was selected from WT, fat free mass (FFM), ideal body weight (IBW), ABW, and a more complex model based on FFM with an additional parameter estimating the relative contribution of fat weight (WT-FFM) on each PK parameter (35). Once the most appropriate size covariate was selected, relationships between model parameters and potential covariates were assessed through inspection of scatterplots and boxplots and evaluated within NONMEM. Covariates included the size measures described above, height, waist circumference, hip circumference, ultrasonographic estimates of subcutaneous adipose tissue, dose in milligrams per kilogram, and total dose. Continuous (linear, power, and exponential) and categorical relationships were assessed (Supplementary Information). Selection of a covariate relationship required a significance of *P* < 0.01, a reduction in IIV, and biological plausibility.

The model evaluation started with the inspection of goodness-of-fit plots including observed versus individual- and population-predicted values, and time versus CWRES. Bootstrapping was performed using Perl-speaks-NONMEM (PSN) with 1,000 samples to obtain 95% empirical confidence intervals for model parameters. Simulation-based model evaluation utilized prediction-corrected visual predictive checks using 1,000 data sets simulated from the final models. To assess the predictive performance of the model and to evaluate any major bias, the observed 10th, 50th, and 90th percentiles and the fraction of samples below the limit of quantification data observed were plotted with their respective simulated 90% *CIs*. Shrinkage of population variability parameters and residual variability was calculated.

### Simulations

Once a final population pharmacokinetic model was established, simulations were performed based on weight and BMI data from the CDC for 20-year-olds (36); 1,000 simulated individuals (equal male and female) were used with the correlation between weight and BMI set at 0.9 (37).

Penicillin concentrations were determined every 6 hours for all simulations. Four-weekly doses of 1.2 MIU of BPG given IM at steady state (12) were simulated and compared to 13-weekly doses of SCIP at the highest investigated dose (10.8 MIU). The results were summarized in terms of time above 20 ng/mL, the commonly regarded target trough level, as well as an exploratory lower target of 10 ng/mL.

## SUPPLEMENTAL MATERIAL

Supplemental file 1 aac.00962-23-s0001.docx Supplemental material.

ASM does not own the copyrights to Supplemental Material that may be linked to, or accessed through, an article. The authors have granted ASM a non-exclusive, world-wide license to publish the Supplemental Material files. Please contact the corresponding author directly for reuse.
