# Supplementary material for: Subcutaneous infusion of high-dose benzathine penicillin G is safe, tolerable, and suitable for less-frequent dosing for rheumatic heart disease secondary prophylaxis: a phase 1 open-label population pharmacokinetic study
Source: Antimicrob Agents Chemother. 2023 Nov 16;67(12):e00962-23. doi: 10.1128/aac.00962-23 (PMC10720493; doi:10.1128/aac.00962-23)
Supplement: Supplemental file 1 — Supplemental material. [file aac.00962-23-s0001.docx]

**Subcutaneous infusion of high-dose benzathine penicillin G is safe, tolerable and suitable for less-frequent dosing for rheumatic heart disease secondary prophylaxis:** **a phase 1, open-label population pharmacokinetic study.**

Joseph Kado^a,b #^, Sam Salman^a,b,c^, Thel K. Hla^a,b,d^, Stephanie Enkel^a,b^, Robert Henderson^e^, Robert M. Hand^a,f^, Adam Hort^g^, Madhu Page-Sharp^h^, Kevin Batty^h^, Brioni R. Moore^a,b,h^, Julie Bennett^i^, Anneka Anderson^j^, Jonathan Carapetis^a,b,l^, Laurens Manning^a,b,d #^

^a^Wesfarmers Centre for Vaccines and Infectious Diseases, Telethon Kids Institute, University of Western Australia, Perth, WA, Australia

^b^Medical School, University of Western Australia, Perth, WA, Australia

^c^Clinical Pharmacology and Toxicology Unit, PathWest, WA, Australia.

^d^Department of Infectious Diseases, Fiona Stanley Hospital, Perth, WA, Australia

^e^Medical Imaging Department, Perth Children’s Hospital, Nedlands, WA, Australia

^f^Department of Infectious Diseases, Royal Perth Hospital, Perth, WA, Australia

^g^Western Australian Country Health Service, Perth, WA, Australia

^h^Curtin Medical School, Curtin University, Bentley, WA, Australia

^i^Department of Public Health, University of Otago, Wellington, New Zealand

^j^Te Kupenga Hauora Maori, University of Auckland, Auckland, New Zealand

^k^Department of Infectious Diseases, Perth Children’s Hospital, Perth, WA, Australia

Running Head: Subcutaneous infusion of Penicillin for RHD **(**SCIP RHD)

#Address correspondence to

Laurens Manning, [laurens.manning@uwa.edu.au](mailto:laurens.manning@uwa.edu.au) OR

Joseph Kado, [joseph.kado@reserch.uwa.edu.au](mailto:joseph.kado@reserch.uwa.edu.au) OR [joseph.kado@telethonkids.org.au](mailto:joseph.kado@telethonkids.org.au)

Supplemental Information

**Methodology**

*Study infusion*

The prescribed benzathine penicillin G (BPG) dose was transferred to a 30mL syringe (JMS Co. Ltd, Hiroshima, Japan) using a female-female luer-lock connector (B. Braun Australia Pty Ltd, Bella Vista, Australia) and delivered over a period of up to 30 minutes using a spring-driven syringe infusion pump (Springfusor^®^30, Go Medical Industries Pty Ltd., Subiaco, Australia). Two mLs of 1% lignocaine was used to prime a variable flow control device (VersaRate^®^ Plus, EMED Technologies, El Dorado Hills, California, USA) and a 22G subcutaneous (SC) catheter (BD Saf-T-Intima^TM^, BD Medical, Mississauga, Ontario, Canada) via the Y-port and the luer lock syringe with the residual lignocaine kept attached.

With the participant supine and abdomen exposed, an ultrasound examination of the lower abdomen was conducted to measure skin and SC layer measurements. The SC catheter was inserted under sterile conditions into the lower anterior abdomen, lateral to the umbilicus and directed laterally. Placement was documented using ultrasound. The flow control device was used to connect the BPG syringe and SC catheter with the system closed. The residual lignocaine was injected into the infusion site, Y-port capped, and the catheter clamped. The BPG syringe was then coupled to the infusion pump, the catheter unclamped, and the flow-regulator adjusted to allow a flow rate between 0.5 and 1.0 mL/min and titrated according to participant tolerance. Prior to removal of the catheter at completion of the infusion, ultrasound confirmed catheter tip position and infusate placement.

*Dried blood spots (DBS)*

The DBS samples collected 3 to 5 drops (10-20μL) of blood, each applied separately, from a fingerprick on special absorbent card (Whatman 903 Protein Saver^TM^ Cards, GE Healthcare, Parramatta, NSW, Australia) which was then folded to allow drying in an airtight container with a 40 gram Hydrosorbent desiccant pack, protected from direct light and either airdried for 1.5-2 hours or stored in a refrigerator at <12^o^C for at least 3 hours. The dried card was the folded to prevent contamination of the DBS, sealed in a foil sachet and stored in -80^o^ freezer for batch assaying at the end of the study.

*Venous sample*

Venous samples for internal validation of the DBS assay were collected at 12-hours and 14-days post-dosing. 4 mLs of whole blood was collected in lithium heparin tube, inverted 4-8 times to prevent coagulation and transported cooled (2-8 ^o^C) to the laboratory. A 0.5mL aliquot of whole blood was removed and stored in a 2 mL freezer tube prior to remainder being centrifuged at 2000G for 12 minutes. The supernatant plasma was aliquoted off into a second freezer tube and the remaining cell pellet placed in a third freezer tube. Samples were separated within an hour of collection and stored separately at -80^o^ C. A validated liquid chromatography-mass spectroscopy assay was used to assay penicillin concentrations.

*Population pharmacokinetic analysis*

The general form of continuous covariate relationships included in the population pharmacokinetic model are provided below. Figure S4 demonstrates an example of how changes in covariate values affect PK parameters for each relationship.

Linear:

$$\theta_{i}=\theta_{POP}+ (x_{i}-\tilde{x})\times\theta_{LINEAR}$$

Where $\theta_{i}$ is the individual PK parameter estimate (after covariate effect applied), $\theta_{POP}$ is the population estimate for the PK parameter, $x_{i}$ is the individual value of the covariate, $\tilde{x}$ is the population average value of the covariate and $\theta_{LINEAR}$ is the effect of the linear covariate relationship.

Power:

$$\theta_{i}=\theta_{POP}\times\left( \frac{x_{i}}{\tilde{x}} \right)^{\theta_{POWER}}$$

Where $\theta_{i}$ is the individual PK parameter estimate (after covariate effect applied), $\theta_{POP}$ is the population estimate for the PK parameter, $x_{i}$ is the individual value of the covariate, $\tilde{x}$ is the population average value of the covariate and $\theta_{POWER}$ is the exponent of the power covariate relationship.

Exponential:

$$\theta_{i}=\theta_{POP}\times EXP\left( (x_{i}-\tilde{x})\times\theta_{EXP} \right)$$

Where $\theta_{i}$ is the individual PK parameter estimate (after covariate effect applied), $\theta_{POP}$ is the population estimate for the PK parameter, $x_{i}$ is the individual value of the covariate, $\tilde{x}$ is the population average value of the covariate and $\theta_{EXP}$ is the effect of the exponential covariate relationship.

**Supporting Information Figures and Tables**

**
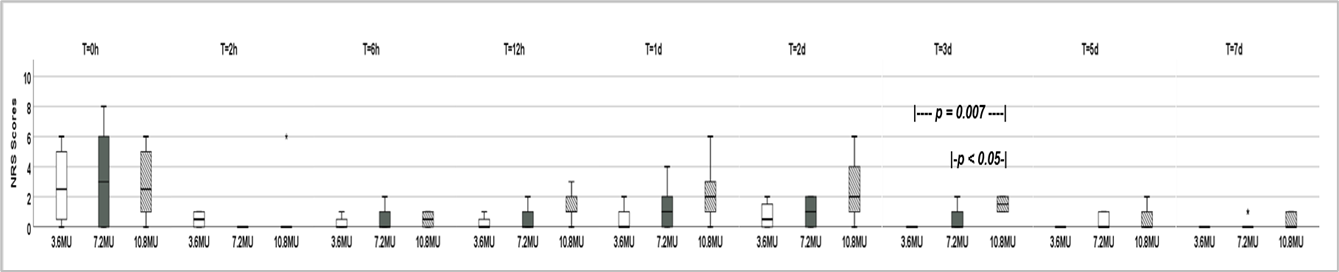
**

**Figure S1.** Comparison of median reported NRS pain scores by time post-infusion and dosing cohort. Using independent-samples median tests, only comparisons for Day 3 (T=3d) demonstrated statistically significant differences in median reported pains scores between the 3.6 and 10.8MIU dose cohorts and the 7.2 and 10.8MIU cohorts with Bonferroni adjusted p values of 0.007 and <0.05 respectively.

**
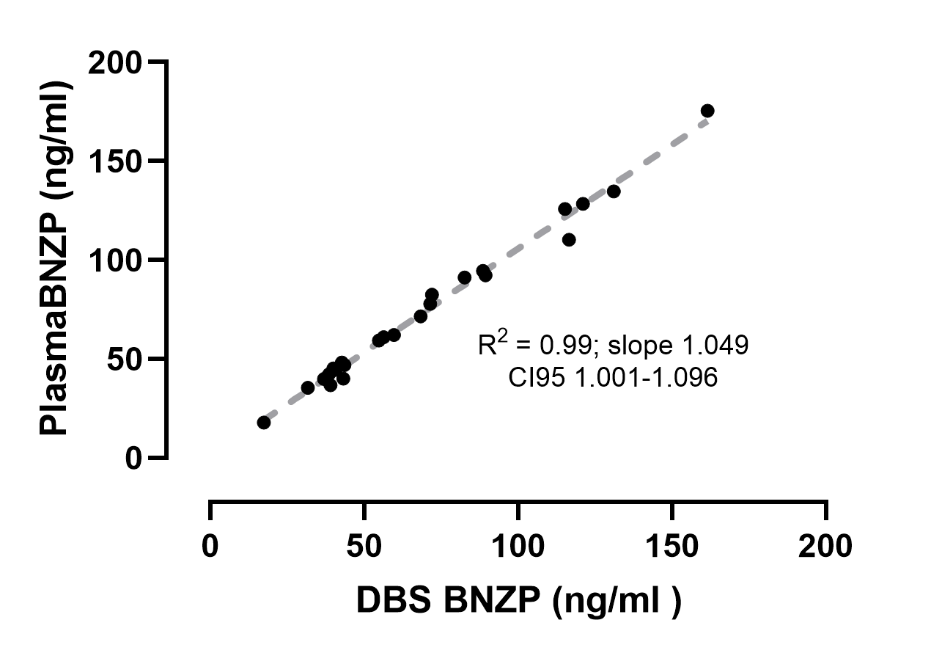
**

**Figure S2.** Comparison between penicillin G concentrations measured from dried blood spots (DBS BNZP) and plasma (PlasmaBNZP). Correlation demonstrated by fitted line (grey dashed) (R^2^ = 0.99, slope 1.049 [95% CI 1.001 – 1.096) overlying individual samples (black dots, n=24).


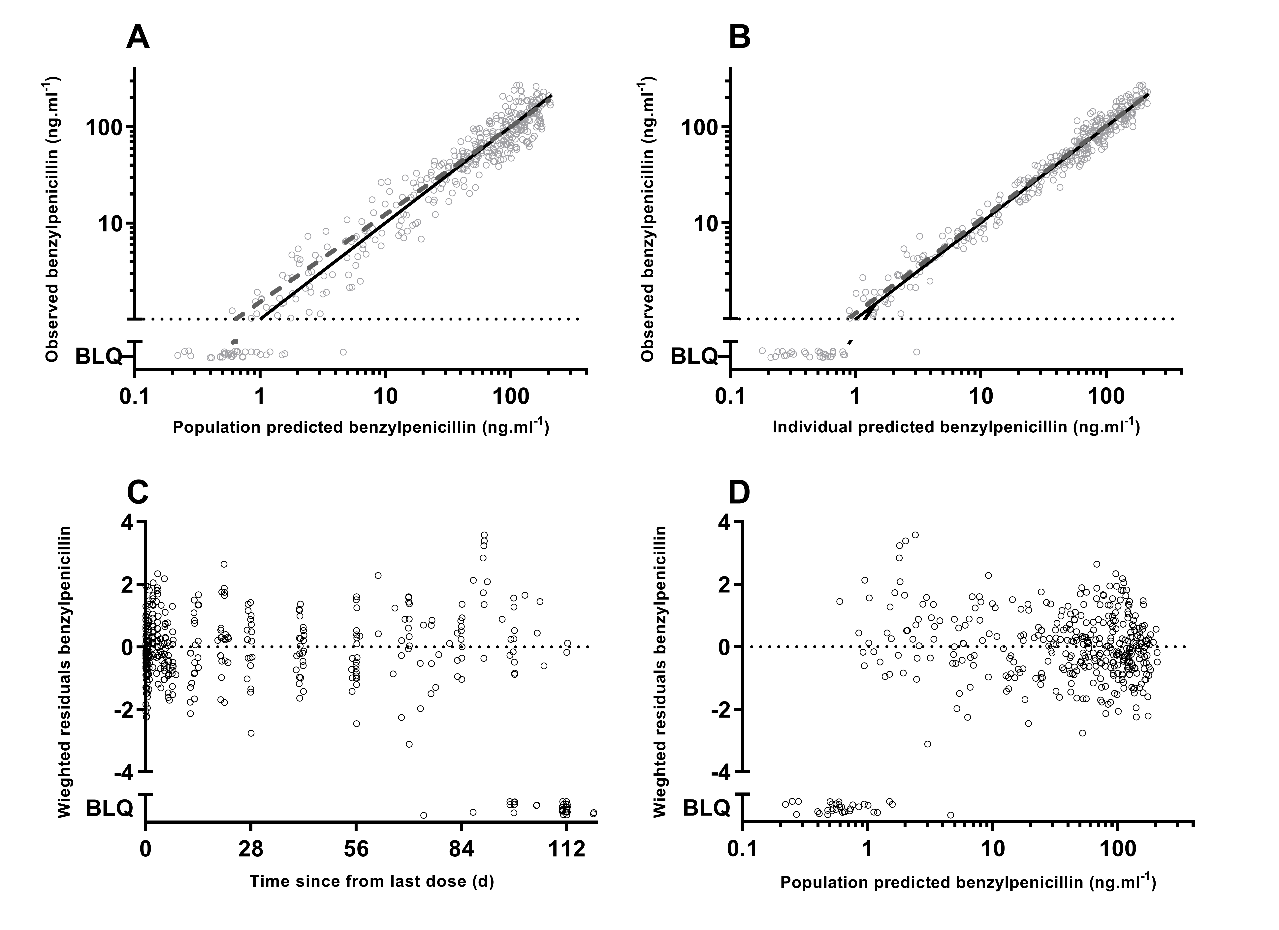


**Figure S3:** Diagnostic plot of the final population model. Observed versus population predicted plasma concentrations (A), observed versus individual predicted plasma concentrations (B), weighted residuals versus time (C), weighted residuals versus population predicted concentrations (D). The solid lines are lines of identity.


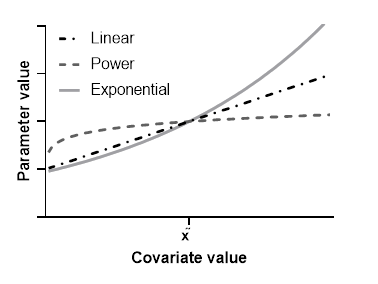


**Figure S4**. Example demonstrating how changes in covariate values affect PK parameters if a linear (black, dot dash line), power (grey, dashed line) or exponential (grey, solid line) relationships is used in the model.

**Table S1.** Comparison of median reported pain scores by BMI group.

| Time post-infusion | Ideal BMI  Median (IQR) | Higher BMI  Median (IQR) | p-value ^a^ |
| --- | --- | --- | --- |
| 0 hours | 2.0 (0.75 - 5.25) | 4.0 (1 - 6) | 0.44 |
| 2 hours | 0 (0 - 0) | 0 (0 - 0) | 0.71 |
| 6 hours | 0.5 (0 - 1) | 0 (0 - 1) | 0.55 |
| 12 hours | 1.0 (0 - 1.25) | 0.5 (0 - 1) | 0.71 |
| 1 day | 0.5 (0 - 1) | 2.0 (1 - 6) | 0.16 |
| 2 days | 1.0 (0 - 2) | 2.0 (0.75 - 2.25) | 0.41 |
| 3 days | 1.0 (0 - 1) | 1.0 (0-2) | 0.55 |
| 5 days | 0 (0 - 0) | 0 (0 - 1) | 0.29 |
| 7 days | 0 (0 - 0) | 0 (0 - 1) | 0.09 |

^a^ Independent samples Mann-Whitney U Test. BMI, body mass index; NRS, numerical rating scale; IQR, interquartile range.

**Table S2. N**ormalised domain and overall Skindex-16 scores by dosing cohort for the first 7 days

|  | **Day 1** | | | | **Day 2** | | | | **Day 3** | | | | **Day 5** | | | | **Day 7** | | | |
| --- | --- | --- | --- | --- | --- | --- | --- | --- | --- | --- | --- | --- | --- | --- | --- | --- | --- | --- | --- | --- |
|  | S | E | F | **Overall Score** | S | E | F | **Overall Score** | S | E | F | **Overall Score** | S | E | F | **Overall Score** | S | E | F | **Overall Score** |
| **Cohort 1** | | | | | | | | | | | | | | | | | | | | |
| Median | 6.3 | 2.4 | 0.0 | **3.4** | 10.4 | 2.4 | 3.3 | **7.1** | 2.1 | 0.0 | 0.0 | **0.7** | 0.0 | 0.0 | 0.0 | **0.0** | 0.0 | 0.0 | 0.0 | **0.0** |
| Min | 4.2 | 0.0 | 0.0 | **1.4** | 4.2 | 0.0 | 0.0 | **2.2** | 0.0 | 0.0 | 0.0 | **0.0** | 0.0 | 0.0 | 0.0 | **0.0** | 0.0 | 0.0 | 0.0 | **0.0** |
| Max | 12.5 | 4.8 | 3.3 | **5.8** | 20.8 | 7.1 | 20.0 | **11.8** | 8.3 | 0.0 | 0.0 | **2.8** | 4.2 | 2.4 | 0.0 | **2.2** | 0.0 | 2.4 | 0.0 | **0.8** |
| UQ | 9.4 | 3.0 | 0.8 | **5.0** | 14.6 | 3.6 | 7.5 | **9.6** | 5.2 | 0.0 | 0.0 | **1.7** | 1.0 | 0.6 | 0.0 | **0.5** | 0.0 | 0.6 | 0.0 | **0.2** |
| LQ | 4.2 | 1.8 | 0.0 | **2.0** | 7.3 | 1.8 | 2.5 | **4.5** | 0.0 | 0.0 | 0.0 | **0.0** | 0.0 | 0.0 | 0.0 | **0.0** | 0.0 | 0.0 | 0.0 | **0.0** |
| **Cohort 2** | | | | | | | | | | | | | | | | | | | | |
| Median | 14.6 | 7.1 | 11.7 | **15.0** | 16.7 | 17.9 | 31.7 | **22.3** | 14.6 | 14.3 | 20.0 | **18.2** | 8.3 | 3.6 | 5.0 | **5.6** | 0.0 | 1.2 | 1.7 | **1.3** |
| Min | 4.2 | 0.0 | 0.0 | **1.4** | 4.2 | 0.0 | 0.0 | **1.4** | 0.0 | 0.0 | 0.0 | **1.4** | 0.0 | 0.0 | 0.0 | **0.0** | 0.0 | 0.0 | 0.0 | **0.0** |
| Max | 70.8 | 52.4 | 53.3 | **56.6** | 75.0 | 54.8 | 60.0 | **61.0** | 66.7 | 23.8 | 46.7 | **45.7** | 33.3 | 14.3 | 20.0 | **22.5** | 16.7 | 9.5 | 16.7 | **11.9** |
| UQ | 22.9 | 25.0 | 49.2 | **23.6** | 20.8 | 23.2 | 51.7 | **30.7** | 20.8 | 16.7 | 33.3 | **19.8** | 11.5 | 6.5 | 12.5 | **10.2** | 8.3 | 4.8 | 9.2 | **7.4** |
| LQ | 8.3 | 3.0 | 5.0 | **9.2** | 9.4 | 14.3 | 6.7 | **12.4** | 5.2 | 7.1 | 5.0 | **6.7** | 0.0 | 2.4 | 0.0 | **0.8** | 0.0 | 0.0 | 0.0 | **0.0** |
| **Cohort 3** | | | | | | | | | | | | | | | | | | | | |
| Median | 14.6 | 13.1 | 16.7 | **15.8** | 10.4 | 10.7 | 8.3 | **9.4** | 4.2 | 2.4 | 3.3 | **3.7** | 4.2 | 2.4 | 0.0 | **2.2** | 0.0 | 2.4 | 0.0 | **0.8** |
| Min | 0.0 | 0.0 | 0.0 | **0.0** | 0.0 | 0.0 | 0.0 | **0.0** | 0.0 | 0.0 | 0.0 | **0.8** | 0.0 | 0.0 | 0.0 | **0.0** | 0.0 | 0.0 | 0.0 | **0.0** |
| Max | 41.7 | 38.1 | 66.7 | **44.6** | 62.5 | 26.2 | 40.0 | **42.9** | 20.8 | 4.8 | 30.0 | **15.8** | 25.0 | 4.8 | 16.7 | **11.9** | 25.0 | 4.8 | 6.7 | **8.3** |
| UQ | 25.0 | 21.4 | 26.7 | **22.8** | 33.3 | 23.8 | 33.3 | **31.0** | 12.5 | 4.8 | 13.3 | **11.6** | 12.5 | 2.4 | 0.0 | **4.2** | 0.0 | 2.4 | 0.0 | **1.6** |
| LQ | 6.3 | 2.4 | 0.0 | **3.4** | 8.3 | 2.4 | 3.3 | **5.5** | 0.0 | 0.0 | 0.0 | **0.8** | 0.0 | 0.0 | 0.0 | **0.8** | 0.0 | 0.0 | 0.0 | **0.0** |
| **Cohorts 1-3** | | | | | | | | | | | | | | | | | | | | |
| Median | 12.5 | 7.1 | 10.0 | **11.1** | 12.5 | 14.3 | 13.3 | **12.5** | 6.3 | 3.6 | 3.3 | **3.7** | 4.2 | 2.4 | 0.0 | **2.2** | 0.0 | 1.2 | 0.0 | **0.8** |
| Min | 0.0 | 0.0 | 0.0 | **0.0** | 0.0 | 0.0 | 0.0 | **0.0** | 0.0 | 0.0 | 0.0 | **0.0** | 0.0 | 0.0 | 0.0 | **0.0** | 0.0 | 0.0 | 0.0 | **0.0** |
| Max | 70.8 | 52.4 | 66.7 | **56.6** | 75.0 | 54.8 | 60.0 | **61.0** | 66.7 | 23.8 | 46.7 | **45.7** | 33.3 | 14.3 | 20.0 | **22.5** | 25.0 | 9.5 | 16.7 | **11.9** |
| UQ | 21.9 | 17.9 | 31.7 | **22.8** | 22.9 | 22.0 | 40.0 | **29.4** | 16.7 | 8.9 | 20.0 | **16.5** | 9.4 | 3.0 | 2.5 | **7.7** | 8.3 | 2.4 | 3.3 | **6.2** |
| LQ | 4.2 | 2.4 | 0.0 | **4.5** | 8.3 | 2.4 | 3.3 | **5.0** | 4.2 | 0.0 | 0.0 | **2.0** | 0.0 | 0.0 | 0.0 | **0.6** | 0.0 | 0.0 | 0.0 | **0.0** |
| *S = symptoms, E = emotions, F = function, UQ = upper quartile, LQ = lower quartile, min = minimum, max = maximum* | | | | | | | | | | | | | | | | | | | | |

**Table S3.** Comparison of normalised overall Skindex-16 scores between Cohort 1 and Cohort 2 by post-infusion day for the first 7 days.

| Post-infusion Day | Cohort 1  Median (IQR) | Cohort 2  Median (IQR) | p-value ^a^ |
| --- | --- | --- | --- |
| Day 1 | 3.4 (2.0-5.0) | 15 (9.2-23.6) | 0.122 |
| Day 2 | 7.1 (4.5-9.6) | 22.3 (12.4-30.7) | 0.307 |
| Day 3 | 0.7 (0-1.7) | 18.2 (6.7-19.8) | 0.007 |
| Day 5 | 0 (0-0.5) | 5.6 (0.8-10.2) | 0.113 |
| Day 7 | 0 (0-0.2) | 1.3 (0-7.4) | 0.320 |

^a^ Independent Kruskal-Wallis Test, significance values adjusted by Bonferroni correction for multiple tests.

**Table S4.** Comparison of overall Skindex-16 scores between ideal- and high-BMI groups

| Post-infusion Day | Ideal BMI  Overall score (IQR) | High BMI  Overall score (IQR) | p-value^a^ |
| --- | --- | --- | --- |
| Day 1 | 9.7 (5.3-20.6) | 12.9 (4.1-37.2) | 0.478 |
| Day 2 | 12.5 (7.6-25.3) | 14.2 (4.6-40.0) | 0.630 |
| Day 3 | 3.1 (1.8-17.0) | 4.0 (2.6-16.6) | 0.590 |
| Day 5 | 0.8 (0.4-3.2) | 2.9 (2.0-9.0) | 0.089 |
| Day 7 | 0 (0-1.2) | 3.4 (0.6-7.3) | 0.060 |
| ^a^ Independent-Samples Mann-Whitney U Test. Significance level is 0.05. | | | |

**Table S5.** Summary of reported adverse events

| **Adverse event** | **Number** | **Duration range (days)** |
| --- | --- | --- |
| Infusion site reaction | 34 | <1-217 |
| US changes | 24 | 111-231 |
| Viral URTI/infections | 11 | 3-19 |
| Headache | 10 | 1-4 |
| Musculoskeletal symptoms | 8 | 1-50 |
| Gastrointestinal symptoms | 3 | 0-27 |
| Others | 11 | <1-90 |
| **Total** | 101 |  |

**Table S6.** Study inclusion and exclusion criteria

| *Study inclusion criteria* |
| --- |
| Participants who meet **all** the inclusion criteria are eligible to be a participant in the trial:   1. Male and females aged 18 - 65 years at the time of screening. 2. BMI between 20kg/m^2^ and 34.9kg/m^2^. 3. No history of chronic renal impairment or significant liver dysfunction. 4. No prior documented allergy to penicillin, cephalosporin antibiotics. 5. Participants who are considered likely to adhere to the trial guidelines for the duration of the trial. 6. Sign and dated informed consent in accordance with Good Clinical Practice (GCP)/Declaration of Helsinki (DoH, Appendix 2). 7. Participants must be in good state of health in the opinion of the investigator as indicated by a comprehensive clinical assessment (medical history and physical exam) and laboratory investigations (haematology, clinical chemistry, and urinalysis). |
| *Study exclusion criteria* |
| Participants who meet **any** of these criteria are not eligible for participation in the trial:   1. Currently taking penicillins or use of any penicillin-based antibiotics from screening through to the final study visit. The use of probenecid, NSAIDs, or other medications which may significantly alter the Bicillin® L-A PK will also not be permitted within 14 days prior to study drug administration until completion of the final follow-up visit. Sporadic NSAID use (<5 occasions) in the 14 days prior to drug administration will be allowed, but the need for ongoing regular NSAIDS will render the person ineligible due to NSAIDS effects on proximal tubular penicillin excretion. Hormonal contraceptives for females and occasional paracetamol and ibuprofen use is permitted while on study. 2. Known soy allergy. 3. History of adverse drug reaction or hypersensitivity. 4. History of seizure disorder 5. Receipt of an investigational product within 3 months of dosing. 6. Planned participation in another clinical trial concurrently. 7. Pregnant or breastfeeding females. 8. Existing dermatological conditions that may affect skin integrity at the site of injection. 9. Planned operation/absence from the study site during the duration of the study. 10. History within the last 12 months of intramuscular, or subcutaneous injection of the abdominal wall, or history of surgery to the buttocks, abdomen or abdominal wall within the last 12 months. 11. History of radiotherapy. 12. Use of any prescription medication or over-the-counter medication, herbal products, vitamins or minerals, within 7 days prior to study drug administration until completion of the final follow-up visit, unless in the opinion of the Principal Investigator or delegate the medication will not compromise participant safety or interfere with study procedures or data validity. 13. Participants who are smokers must abstain from using tobacco products during the confinement period. 14. Laboratory tests that fail to meet the following thresholds: one repeat will be allowed at discretion of the investigator to confirm eligibility.     1. Haematology: complete blood count (Haemoglobin, total white cell count and platelet count) – parameters within gender-specific reference intervals from reference laboratory unless deemed not clinically significant by the investigator.     2. Clinical chemistry within gender-specific reference intervals from PathWest unless deemed not clinically significant by the investigator: urea, glucose, creatinine, sodium, potassium, chloride and bicarbonate, lactate dehydrogenase, calcium, total protein, magnesium, phosphate, albumin, cholesterol, and uric acid. For renal function an eGFR >90ml/min/m^2^ will be considered normal using the CKD-EPI without albuminuria on dipstick].     3. Liver function tests (only at screening): aspartate aminotransferase, alanine aminotransferase, alkaline phosphatase, total bilirubin, gamma-glutamyl transferase [<1.5 x ULN (ALT, GGT; PathWest gender-specific reference ranges) will be considered not clinically significant].     4. Negative HIV, Hepatitis B and C serology.     5. Negative pregnancy test at screening and check-in (females). |

**Table S7.** Study schedule of assessments

|  | **Screen** | **Administration period** | | | | | | | | | | | | | | | | | | | **Follow-up Period** |
| --- | --- | --- | --- | --- | --- | --- | --- | --- | --- | --- | --- | --- | --- | --- | --- | --- | --- | --- | --- | --- | --- |
|  | 4 weeks | Day 0 | | | | | Day 1 | Day 2 | Day 3 | Day 5 | Day 7 | Day 14 | Day 21 | Day 28 | Day 42 | Day 56 | Day 70 | Day 84 | Day 98 | Day 112 | Day 126 |
|  |  | Pre-dose | 0 h | 2 | 6 | 12 | 24 | 48 | 72 |  |  |  |  |  |  |  |  |  |  |  |  |
| Sampling window |  | - | - | ± 5m | ± 15m | ± 30m | ±  4h | ± 8h | ± 12h | ± 1d | ±  1d | ±  2d | ±  2d | ±  2d | ±  2d | ±  7d | ±  7d | ±  7d | ±  7d | ±  7d | ±  7d |
| Informed Consent | X |  |  |  |  |  |  |  |  |  |  |  |  |  |  |  |  |  |  |  |  |
| Inclusion/Exclusion Criteria | X | X |  |  |  |  |  |  |  |  |  |  |  |  |  |  |  |  |  |  |  |
| DBS PK Sample |  | X |  | X | X | X | X | X | X | X | X | X | X | X | X | X | X | X | X | X |  |
| Venous PK Samples |  |  |  |  |  | X |  |  |  |  |  | X |  |  |  |  |  |  |  |  |  |
| Pain Score (NRS) |  |  | X | X | X | X | X | X | X | X | X^1^ | X^1^ | X^1^ | X^1^ | X^1^ | X^1^ | X^1^ | X^1^ | X^1^ | X^1^ |  |
| Monitoring of Skin Irritation |  |  |  |  |  |  | X | X | X^1^ | X^1^ | X^1^ | X^1^ | X^1^ | X^1^ | X^1^ | X^1^ | X^1^ | X^1^ | X^1^ | X^1^ |  |
| Ultrasound scan of Injection Site |  | X | X |  |  |  |  |  |  |  |  |  |  | X |  | X^2^ |  | X^2^ |  | X^2^ |  |
| Qualitative questionnaire |  |  | X | X |  |  |  |  |  |  | X |  |  |  |  |  |  |  |  |  |  |
| IP Administration |  |  | X |  |  |  |  |  |  |  |  |  |  |  |  |  |  |  |  |  |  |
| Adverse Events |  |  |  |  |  |  |  |  |  |  |  |  |  |  |  |  |  |  |  |  |  |
| In Clinic Confinement |  |  |  |  |  |  |  |  |  |  |  |  |  |  |  |  |  |  |  |  |  |
| Telephone Call Follow-up |  |  |  |  |  |  |  |  |  |  |  |  |  |  |  |  |  |  |  |  | X |

*^1^ Assessments performed only if pain or irritation was recorded at the previous visit. ^2^ Assessments performed only if ultrasound changes were identified previously. DBS-dried blood spots, PK-pharmacokinetic, NRS- numerical rating scale, IP- investigative product, m- minutes, h-hours, d- days.*

**Table S8.** SCIP study Modified Skindex-16 Questionnaire for semi-qualitative assessment of the infusion site reaction.

| *How often have you been bothered by:* | | | | | | | | |
| --- | --- | --- | --- | --- | --- | --- | --- | --- |
|  |  | Never bothered |  |  |  |  |  | Always bothered |
| 1 | Your injection site itching | _0_ | _1_ | _2_ | _3_ | _4_ | _5_ | _6_ |
| 2 | Your injection site burning or stinging | _0_ | _1_ | _2_ | _3_ | _4_ | _5_ | _6_ |
| 3 | Your injection site hurting | _0_ | _1_ | _2_ | _3_ | _4_ | _5_ | _6_ |
| 4 | Your injection site being irritated | _0_ | _1_ | _2_ | _3_ | _4_ | _5_ | _6_ |
| 5 | The persistence/reoccurrence of your injection site | _0_ | _1_ | _2_ | _3_ | _4_ | _5_ | _6_ |
| 6 | Worry about your injection site | _0_ | _1_ | _2_ | _3_ | _4_ | _5_ | _6_ |
| 7 | The appearance of your injection site | _0_ | _1_ | _2_ | _3_ | _4_ | _5_ | _6_ |
| 8 | Frustration about your injection site | _0_ | _1_ | _2_ | _3_ | _4_ | _5_ | _6_ |
| 9 | Embarrassment about your injection site | _0_ | _1_ | _2_ | _3_ | _4_ | _5_ | _6_ |
| 10 | Being annoyed about your injection site | _0_ | _1_ | _2_ | _3_ | _4_ | _5_ | _6_ |
| 11 | Feeling depressed about your injection site | _0_ | _1_ | _2_ | _3_ | _4_ | _5_ | _6_ |
| 12 | The effects of your injection site on your interactions with others | _0_ | _1_ | _2_ | _3_ | _4_ | _5_ | _6_ |
| 13 | The effects of your injection site on your desire to be with people | _0_ | _1_ | _2_ | _3_ | _4_ | _5_ | _6_ |
| 14 | Your injection site makes it hard to show affection | _0_ | _1_ | _2_ | _3_ | _4_ | _5_ | _6_ |
| 15 | The effects of your injection site on your daily activities | _0_ | _1_ | _2_ | _3_ | _4_ | _5_ | _6_ |
| 16 | Your injection site making it hard to work or do what you enjoy | _0_ | _1_ | _2_ | _3_ | _4_ | _5_ | _6_ |
